# Soil bacterial community composition is altered more by soil nutrient availability than pH following long-term nutrient addition in a temperate steppe

**DOI:** 10.3389/fmicb.2024.1455891

**Published:** 2024-09-13

**Authors:** Hao Zhang, Na Jiang, Siyu Zhang, Xiaoyu Zhu, Hui Wang, Weiming Xiu, Jianning Zhao, Hongmei Liu, Haifang Zhang, Dianlin Yang

**Affiliations:** ^1^Agro-Environmental Protection Institute, Ministry of Agriculture and Rural Affair, Tianjin, China; ^2^State Key Laboratory of Environmental Criteria and Risk Assessment, Chinese Research Academy of Environmental Sciences, Beijing, China

**Keywords:** nutrient addition, bacterial diversity, nutrient cycling, bacterial community composition, temperate steppe

## Abstract

Although aboveground biodiversity has been extensively studied, the impact of nutrient enrichment on soil microbial populations remains unclear. Soil microorganisms serve as important indicators in shaping soil nutrient cycling processes and are typically sensitive to nutrient additions. For this, we employed a factorial combination design to examine the impact of nutrient additions on the composition and function of soil bacteria in a temperate steppe. Nitrogen addition promoted the growth of copiotrophic bacteria (Proteobacteria, Firmicutes, and Bacteroidota) but inhibited the growth of oligotrophic bacteria (Acidobacteria, Chloroflexi, and Verrucomicrobiota). Phosphorus addition alleviated phosphorus deficiency, resulting in a decrease in the abundance of phoD-harboring bacteria (Actinobacteria and Proteobacteria). Significant enhancement of soil bacterial alpha diversity was observed only in treatments with added phosphorus. Changes in NO_3_^−^-N, NH_4_^+^-N, available phosphorus, and dissolved organic carbon resulting from nutrient addition may have a greater impact on microbial community structure than changes in soil pH caused by nitrogen addition. Moreover, nutrient addition may indirectly impact microbial ecological function by altering nutrient availability in the soil. In conclusion, our study suggests that soil nutrient availability, particularly available phosphorus, affects soil bacterial communities and potentially regulates the biogeochemical cycles of soil ecosystems.

## 1 Introduction

Natural or semi-natural grasslands have higher biodiversity and lower spatial homogeneity compared to intensively managed cropland (Fan et al., 2018; Peng et al., 2024). However, grassland degradation has been accelerating due to the growth of livestock production driven by the ever-increasing demand for ruminant meat and milk (Bouwman, 2013; Sattari et al., 2016). This will lead to a sustained reduction in soil fertility because grass harvesting or grazing removes nutrients from the ecosystem (Zhang et al., 2022). Nutrient additions are common management activities aimed at maintaining the sustainability of grassland production and increasing the input of limiting nutrients into grassland ecosystems (Mayel et al., 2021). Supplementing nutrients compensates for soil nutrient loss and maintains nutrient availability at optimal levels. Generally, the introduction of limiting nutrients (e.g., N and P) results in rapid biomass growth, coupled with a decrease in plant diversity (Elser et al., 2007; Fang et al., 2023). Previous research has mainly focused on changes in aboveground plant biomass and diversity to understand ecosystem feedback to nutrient enrichment (Chen et al., 2022). Mounting evidence suggests that soil microorganisms play a key role in maintaining ecosystem multifunctionality and preserving soil fertility by performing almost all soil biochemical processes (Leff et al., 2015). Soil microbes serve as both a source and a sink of readily available nutrients (Li et al., 2015).

The transformation of soil nutrients depends on soil microorganisms, which are often sensitive to nutrient inputs (Chen et al., 2022; Yan et al., 2022). Previous studies have focused on exploring how single nutrient addition (e.g., N and P) influences soil bacterial community composition (Wang et al., 2018a; Zhang et al., 2019). These studies have yielded mixed results, with alterations in community composition and taxonomy not consistently trending in the same direction or extent with the addition of single nutrients (Fierer et al., 2012; Leff et al., 2015; Prober et al., 2015). These variations could be explained by factors, such as vegetation composition, environmental conditions, and climate, but might also indicate that the impact on soil bacterial communities is influenced by the combined addition of mineral nutrients (Egan et al., 2018). For example, the addition of P and K together, as well as N-only addition, can significantly change the composition of soil bacterial communities (Cuhel et al., 2019; Sarula Yang et al., 2022). Dai et al. (2018) showed that multi-nutrient (N, P, and K) additions could mitigate the adverse effects of adding N-only on bacterial communities. Recent studies are increasingly focusing on how nutrient additions influence microbial communities in soil. In addition to climatic factors, soil pH is a crucial determinant of soil microbial community composition and function on both global and regional scales (Fierer and Jackson, 2006; Liu et al., 2020). Most bacteria typically exhibit optimal growth at neutral pH values, and soil acidification induced by N addition often leads to a reduction in soil bacterial diversity (Dai et al., 2018). A previous study found that pH significantly influences changes in soil bacterial communities, with an average decrease of 0.16 units in soil pH at 25 grassland sites when N was added (Leff et al., 2015). In addition, there are differences in the life history strategies of soil microorganisms, such as the response to nutrients, which can be categorized into oligotrophic and copiotrophic bacteria (Fierer et al., 2007; Stone et al., 2023). Consequently, changes in soil nutrient availability resulting from nutrient addition may further impact the structure and metabolic processes of soil microbial communities (Wang et al., 2017; Xiao et al., 2022). Previous studies have also indicated that soil bacterial communities may be affected by variations in nutrient availability and plant-derived carbon resulting from nutrient additions (Philippot et al., 2013; Wang et al., 2018a).

Hulunbuir grassland is located in the east of Eurasian Steppes, building an ecological security barrier in northeast China. However, land degradation is a prevalent issue in this region, primarily driven by climate conditions and the growth of livestock production. Zhang et al. (2018) discovered a potential mechanism for plant–microbe–soil interactions regarding nitrogen deposition and precipitation change in this region. Therefore, we examine whether and how soil bacterial community composition and functional traits have been influenced by different nutrient addition treatments compared to grasslands with no nutrient addition. Based on the existing findings, we hypothesized the following: (1) soil bacteria are more sensitive to long-term nitrogen addition than to phosphorus addition and that the decline in soil pH resulting from nitrogen addition is associated with a decrease in soil bacterial diversity and (2) the changes in the soil bacterial community structure and functions are induced by altered soil pH and nutrient availability.

## 2 Materials and methods

### 2.1 Study site and soil sampling

This study took place at a typical location in the *Stipa Baicalensis* steppe (119°42′E, 48°30′N) in Hulunbuir, Inner Mongolia. A temperate continental monsoon climate is a characteristic of this region, with warm summers and cold winters. The annual rainfall is 329 mm, and the average temperature is −0.7°C. The vegetation is a typical temperate meadow steppe dominated by *S. baicalensis* and *Leymus chinensis*. This region had never been fertilized before 2010.

In June 2010, a comprehensive factorial experiment involving N, P, and K additions was initiated at the study site. It included eight treatment combinations (N, P, K, NP, NK, PK, NPK, and Control) and was designed using a randomized block design. Four blocks (replicates) were established, giving a total of 32 plots. Specifically, nitrogen (as urea), phosphorus (as triple superphosphate), and potassium (as potassium sulfate) fertilizers were applied at a rate of 100 kg ha^−1^. All fertilizer nutrient values were determined by the standard protocol of NutNet (Borer et al., 2014). From 2010 to 2021, the nutrients were added annually in the second week of June.

Soil sampling was performed on 12 August 2021, and soil subsamples were collected with a soil probe from 10 random positions at a depth of 0–20 cm and combined, with 4 replicates per treatment. Each soil sample was passed through a 2 mm sieve. Then, composite samples were divided into two parts: One part was used to measure soil physicochemical properties by natural drying, and the other part was stored at −80°C for later DNA extraction. Soil pH, organic carbon (SOC), total N (TN), total P (TP), inorganic N (NH_4_^+^–N and NO_3_^−^–N), available P (TP), available K (AK), dissolved organic carbon (DOC), and soil water content (SWC) were measured according to previously described methods (Bao, 2000), and the results are shown in Supplementary Table 1.

### 2.2 DNA extraction, amplification, and sequencing

The AxyPrep DNA Gel Extraction Kit (Axygen Biosciences, United States) was used to extract the DNA of each soil sample. The bacterial V3-V4 region of 16S rRNA genes was amplified using primer pairs 338F (5’-ACTCCTACGGGAGGCAGCAG-3′) and 806R (5’-GGACTACHVGGGTWTCTAAT-3′; Yan et al., 2022). Sequencing was performed using the Illumina MiSeq PE 300 platform (Illumina, San Diego, CA, United States) by Majorbio (Shanghai, China). Detailed information on the PCR protocols and subsequent bioinformatics analysis are based on the methods described by Zhang et al. (2024). In total, 1,300,367 (40,637 ± 4,291 on average) high-quality and valid 16S sequences were obtained for the 32 soil samples. After rarefying the same number of sequences, 1,095,008 (34,219 on average) sequences were obtained. Alpha diversity was calculated using the indexes of Shannon, Chao1, observed OTUs, and phylogenetic diversity (PD). Annotations of the ecological functions of C and N cycling were made using the FAPROTAX database on the Majorbio Cloud Platform.^1^ Sequencing data were deposited in the NCBI under the accession number PRJNA928583.

### 2.3 Statistical analyses

All statistical analyses were performed using the R environment v4.2.1 (R Core Team, 2022) unless otherwise stated. One-way ANOVA was used to analyze the soil and bacterial variables. Tukey’s honestly significant difference (HSD) test was then used to evaluate differences between the different nutrient addition treatments, and Student’s *t*-test was employed to determine significant differences between single and multiple additions. Three-way ANOVA was performed using SPSS 23.0 (SPSS, Chicago, IL, United States) to further determine the interaction effects of N, P, and K additions. Principal coordinate analysis (PCoA) and PERMANOVA were performed by the package of “vegan.” The environmental factors significantly correlated with community composition (abundance of OTUs) were identified using a Mantel test of Bray–Curtis similarity distance values, which provided a correlation measure between each variable and the bacterial community structure (r_M_). In the dbRDA analysis, the explanatory variables were analyzed one by one to assess their respective contributions to explaining variance (dbRDA % variance explained). Then, we simplified the model containing all explanatory variables by forward-model selection using the function ordiR2ste (Blanchet et al., 2008). Variance partitioning analysis (VPA) was performed to assess the relative contributions of variables (Lai et al., 2022). Models were developed from the measurements and tested using structural equation modeling (SEM) with regression analysis and previous experience to reveal the effects of soil properties on microbial communities under nutrient addition, using AMOS 22.0 software. Model adequacy was determined using the chi-square (χ^2^) test, comparative fit index (CFI), goodness of fit index (GFI), and root-mean-square error of approximation (RMSEA). Pearson’s correlation analysis between microbial genera and soil properties was performed using the “corr.test” function of the R psych package. The correlation network was visualized using Cytoscape 3.9.1 software.

## 3 Results

1. Effects of nutrient additions on the main phyla and genera of soil bacteria

**Figure 1:**
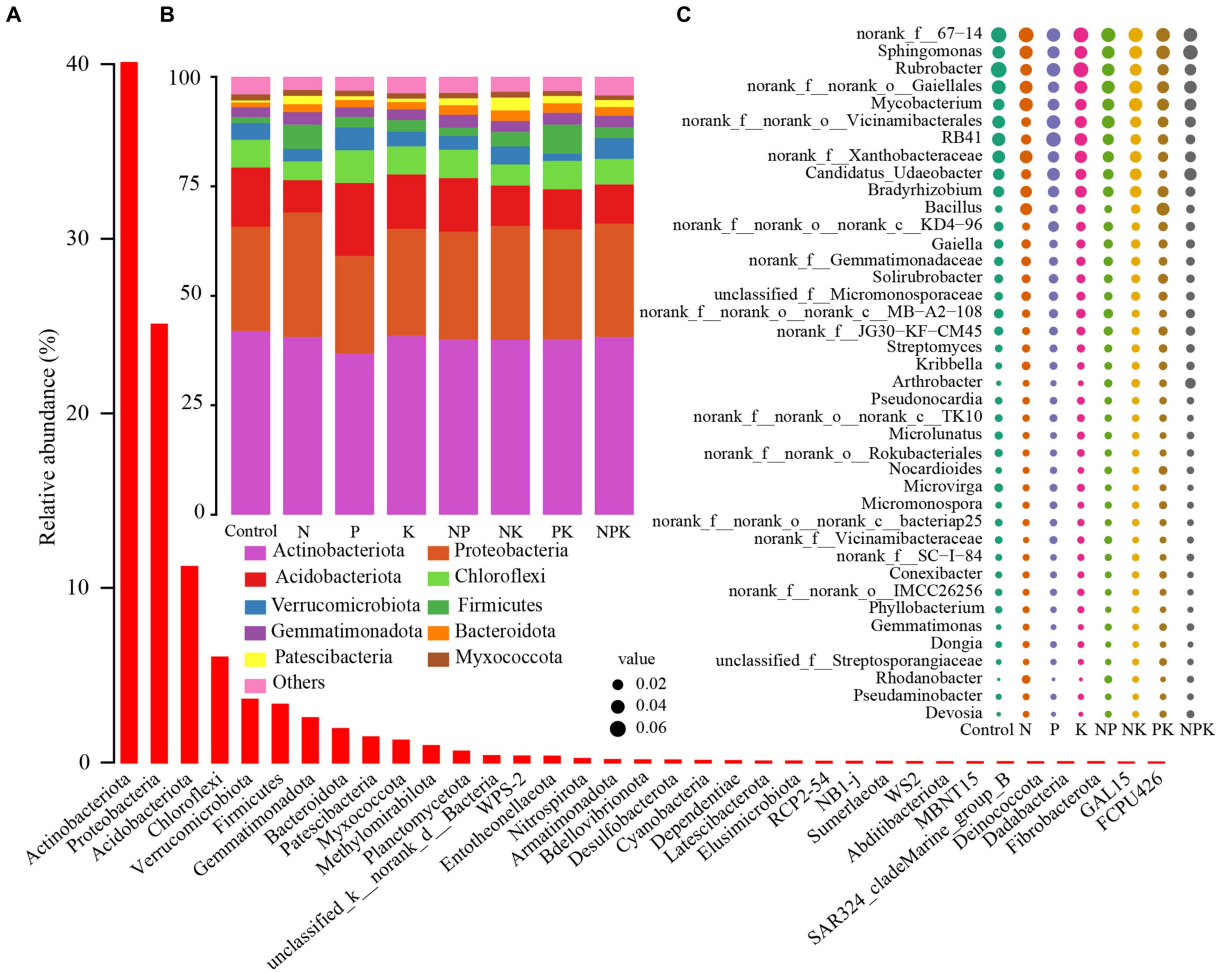
Relative abundances of bacterial phyla and genera under different nutrient addition treatments. Bacteria at the phylum level **(A)**, top 10 bacterial phyla **(B)**, and top 40 genera **(C)** under different nutrient addition treatments.

**Figure 2:**
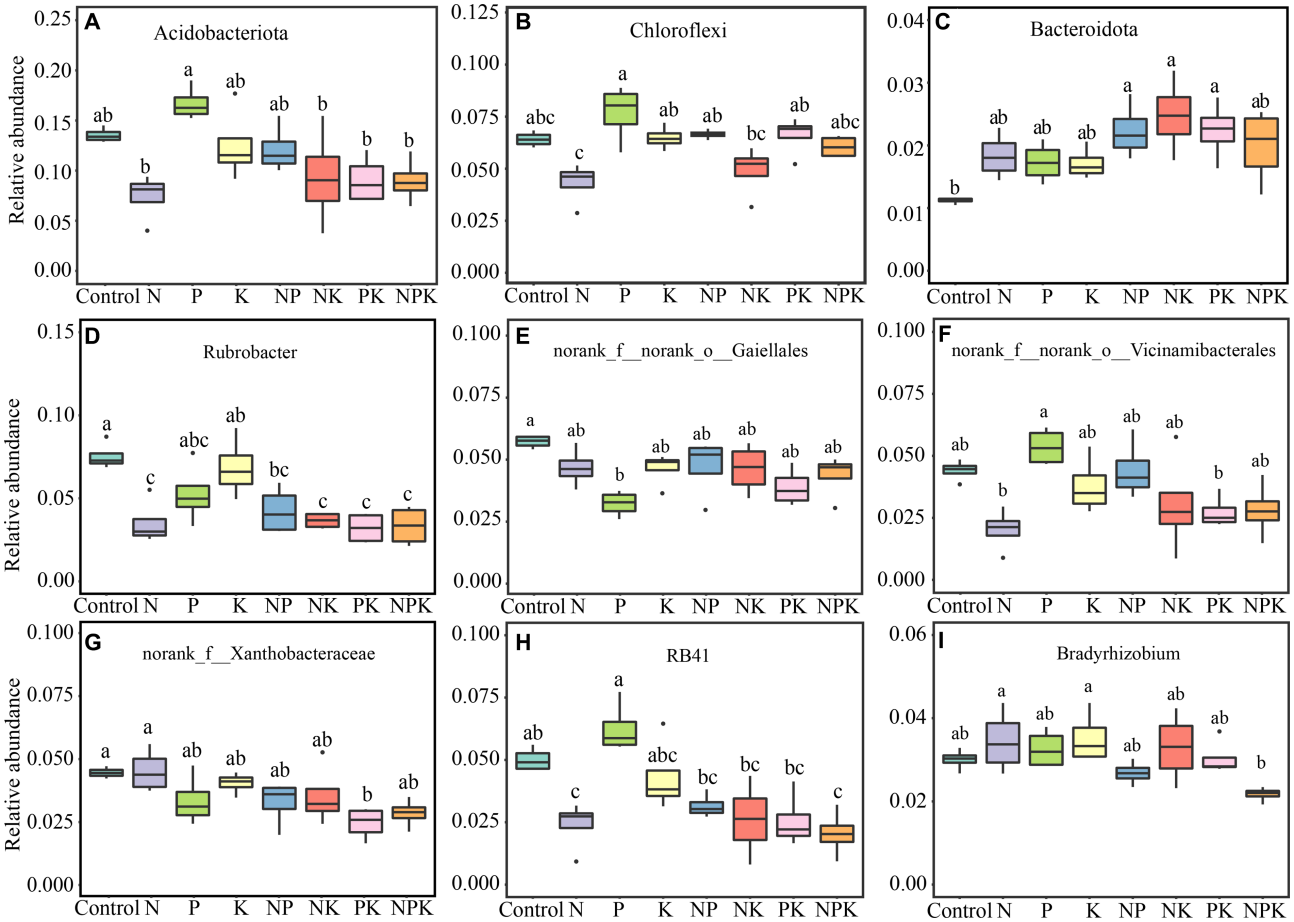
Analysis of significant differences among the top 10 species in the phylum and genus of soil bacteria under different nutrient addition treatments. The significance of differences at the phylum **(A–C)** and genus **(D–I)** levels. Different lowercase letters above each box in the same subfigure indicate significant differences between treatments (Tukey’s HSD test, *p* < 0.05).

The grassland soil environment was composed of 35 phyla for the bacterial community. *Actinobacteriota* (40.07%) was the most abundant phylum, followed by *Proteobacteria* (25.07%), *Acidobacteriota* (11.20%), *Chloroflexi* (6.01%), *Verrucomicrobiota* (3.59%), *Firmicutes* (3.31%), *Gemmatimonadota* (2.54%), *Bacteroidota* (1.92%), *Patescibacteria* (1.44%), *Myxococcota* (1.24%), and other bacterial phyla (3.61%; Figures 1A,B). Among the top 10 most abundant bacterial phyla, *Acidobacteriota*, *Chloroflexi*, and *Bacteroidota* varied significantly (Figure 2). The P-only addition led to an increase in *Acidobacteriota* and *Chlorofle*xi. However, the N-only and NK addition treatments showed an opposite trend, although this effect was not statistically significant (Figures 2A–C). In comparison with the P-only addition, *Acidobacteriota* significantly decreased with the N-only, NK, PK, and NPK addition treatments (Figure 2A), while *Chloroflexi* decreased with the N-only and NK addition treatments (Figure 2B). The abundance of *Bacteroidota* increased with all nutrient addition treatments, rising by 50.13, 54.82, and 49.56% with NP, NK, and PK additions, respectively (Figure 2C). In addition, N and P additions significantly affected multiple bacterial phyla (Supplementary Table 4). Furthermore, our study suggested that *Bacteroidota* and *Patescibacteria* showed significant differences between single nutrient additions and multiple nutrient additions (*p* < 0.05; Supplementary Table 3).

Among those OTUs that could be *classified* into genera, *Sphingomonas* (4.77%) was the most abundant genus, followed by *Rubrobacter* (4.70%), *Mycobacterium* (3.94%), *RB41* (3.55%), *Candidatus_Udaeobacter* (3.09%), *Bradyrhizobium* (3.06%), *Bacillus* (2.13%), Gaiella (1.71%), and other bacterial genera (Figure 1C). In addition, several unranked bacterial genera also exhibited relatively high abundances, including *unranked 67–14* (6.06%), *unranked Gaiellales* (4.42%), *unranked Vicinamibacterales* (3.59%), and *unranked Xanthobacteraceae* (3.54%; Figure 1C). The relative abundances of six genera within the top 10 most abundant bacterial genera were significantly altered by different nutrient addition treatments (Figure 2). N and multiple nutrient additions (NP, NK, PK, and NPK) significantly decreased *Rubrobacter* (Figure 2D; Supplementary Table 3). Moreover, both N and NPK additions significantly decreased *RB41* (Figure 2H), and PK addition significantly decreased *unranked Xanthobacteraceae* (Figure 2G). P addition had a marginally positive effect on *unranked Vicinambacterales* and *RB41* but significantly decreased the *unranked Gaiellales* (Figure 2). N, P, and K additions, along with their interactive effects, significantly affected multiple bacterial genera (Supplementary Table 6). In addition, our study indicated that multiple bacterial genera showed significant differences between single nutrient additions and multiple nutrient additions (Figure 1; Supplementary Table 3).

### 3.1 Effects of nutrient additions on soil bacterial diversity and community structure

We analyzed the differences in bacterial diversity among different nutrient addition treatments. Nutrient addition treatments tended to have higher Shannon diversity, Chao1, and observed OTU richness than the control, but the differences were not significant (Table 1). P addition and the interaction between P and K addition significantly affected multiple diversity indicators (Supplementary Table 2). In addition, P-only addition significantly increased phylogenetic diversity (*p* < 0.05; Table 1), and there was no significant difference in bacterial alpha diversity between single additions and multiple additions (*p* < 0.05; Supplementary Table 3). There was a significant correlation between bacterial diversity and soil chemical properties (TP, AP, NO_3_^−^-N, C/P, and C/N; Supplementary Table 4).

**Table 1:** Soil bacterial α-diversity under different nutrient addition treatments.

Treatments	Shannon	Chao1	PD	Number of observed OTUs
Control	5.95 ± 0.04a	2894.12 ± 89.65a	165.11 ± 3.32b	2122.75 ± 40.84a
N	5.96 ± 0.18a	3050.45 ± 266.62a	170.74 ± 14.46ab	2167.50 ± 220.41a
P	6.15 ± 0.08a	3262.66 ± 30.47a	185.48 ± 5.03a	2394.25 ± 65.74a
K	6.06 ± 0.06a	3236.97 ± 192.96a	179.00 ± 6.15ab	2315.25 ± 50.69a
NP	6.22 ± 0.07a	3171.52 ± 155.73a	182.20 ± 4.59ab	2337.50 ± 88.42a
NK	6.00 ± 0.19a	2958.27 ± 218.06a	170.52 ± 10.37ab	2187.25 ± 129.25a
PK	6.10 ± 0.26a	3199.44 ± 170.64a	183.78 ± 8.47ab	2355.00 ± 161.73a
NPK	6.08 ± 0.10a	2949.64 ± 318.23a	170.93 ± 9.4ab	2173.50 ± 168.04a

Data are presented as mean ± standard deviation (SD). Different labeled letters indicate significant differences between different treatments according to one-way ANOVA with Tukey’s HSD test (*p* < 0.05).

**Figure 3:**
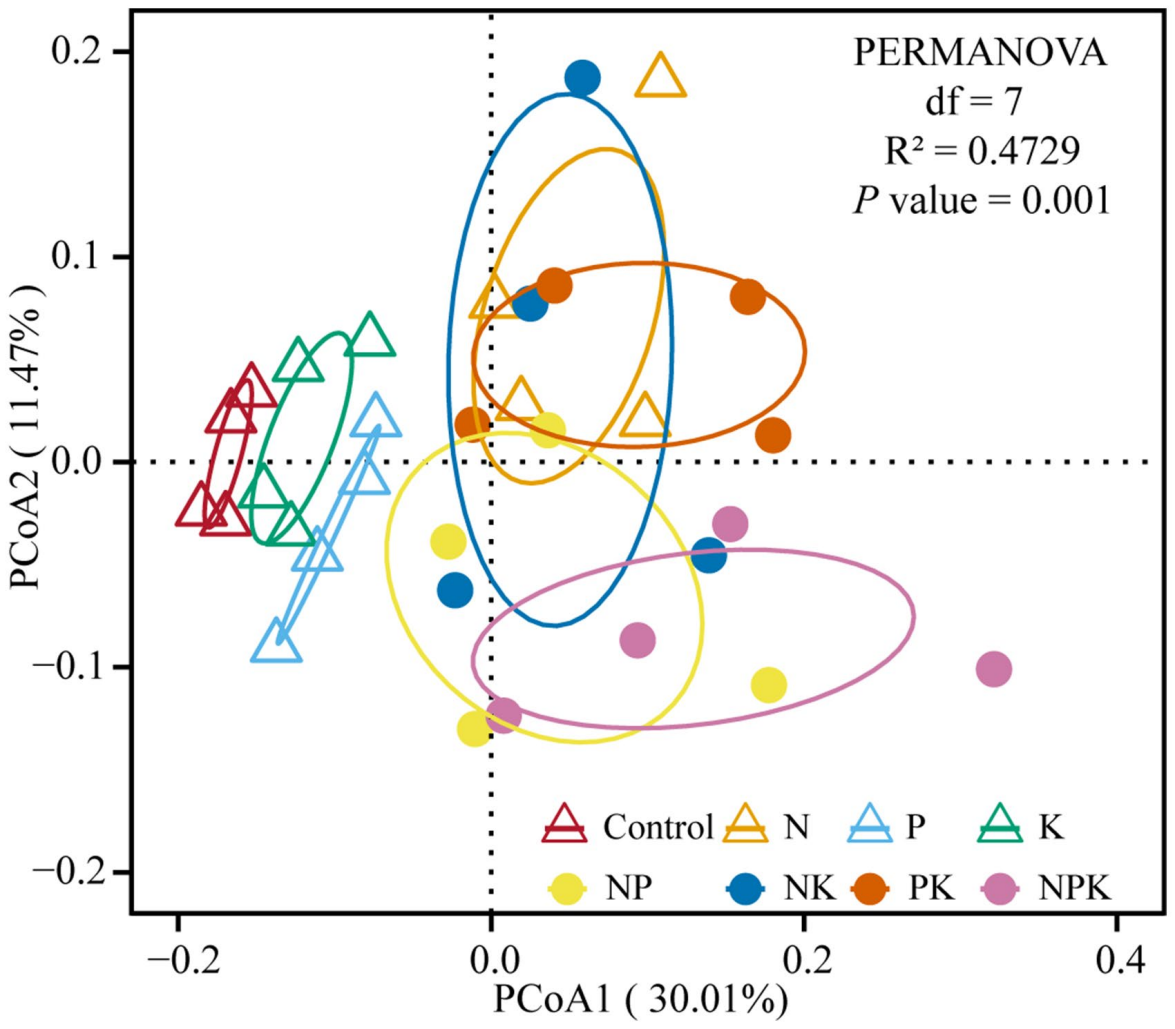
Bacterial community composition in different nutrient addition treatments. PCoA plots based on the Bray–Curtis distance showing the overall distribution pattern of the bacterial community.

PERMANOVA results showed variations in bacterial structure under different nutrient addition treatments (R^2^ = 0.4729, *p* = 0.001; Figure 3). The PCoA showed that samples of the control, P, and K clustered together and were separated from N and multiple nutrient additions (NP, NK, PK, and NPK) along PCoA1. In addition, the samples of N and PK clustered together and were separated from NP and NPK along PCoA2 (Figure 3).

**Figure 4:**
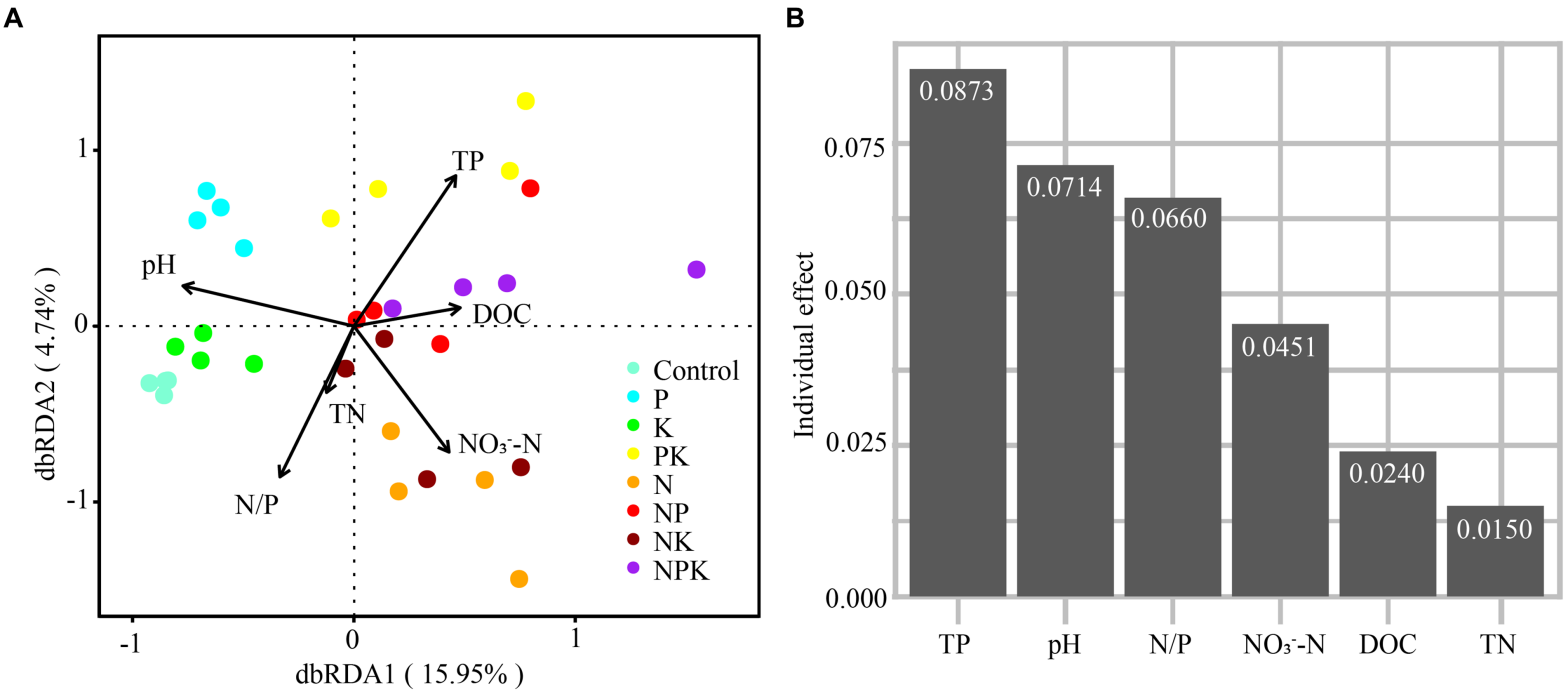
Relationship between environmental factors in the bacterial community. The effects of environmental factors on bacterial community composition by using **(A)** redundancy analysis (RDA) and **(B)** variation partition analysis (VPA).

**Figure 5:**
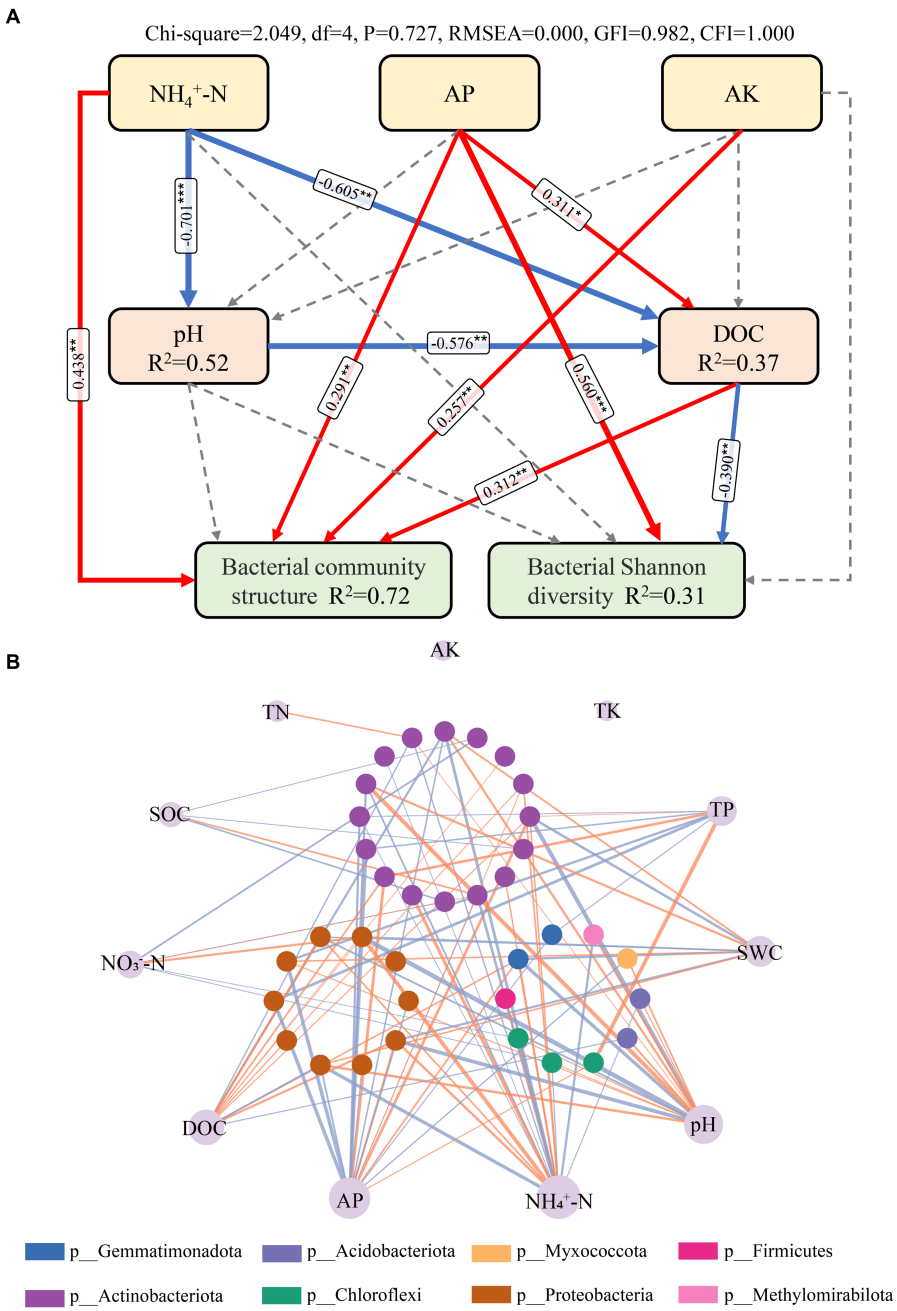
Effects of environmental factors on bacterial community structure in a meadow Steppe. **(A)** Structural equation modeling (SEM) analysis of the effects of nutrient addition on soil bacterial community structure and Shannon diversity via pathways related to soil chemical attributes. Solid red and solid blue arrows indicate positive and negative relationships, respectively. Thickness of the arrows is proportional to the strength of standardized path coefficients. R2 represents the proportion of variance explained. Asterisks indicate the significance level: *p < 0.05, **p < 0.01. **(B)** Correlation network between environmental variables and their directly connected nodes from the entire nutrient addition treatments. Nodes in the outer ring represent different environmental variables, and nodes in the three inner ring represent different bacterial genera. Node size of environmental variables is proportional to node connectivity. Node color in the three inner ring represent various phylogenetic phyla. The line linking nodes represent significant correlations between environmental factors and bacterial genera, with line width varying proportionally to the absolute value of the correlation coefficient. Orange lines indicate positive interactions and purple lines indicate negative interactions.

Among all soil chemical variables, soil pH (13.9%, r_M_ = 0.368) and TP (9.9%, r_M_ = 0.363) were the best individual predictors, followed closely by AP (9.3%, r_M_ = 0.245), NH_4_^+^-N (9.2%, r_M_ = 0.241), and C/P (8.2%, r_M_ = 0.225; Table 2). The distance-based redundancy analysis (dbRDA) based on forward-model selection retained six environmental factors, namely, NO_3_^−^-N, DOC, TP, pH, N/P, and TN, and the results indicate that all factors, except TN, were significantly correlated with the variations in bacterial community structure (Figure 4A). The VPA indicated that all factors together explained 30.9% of the total variation (Figure 4B). Moreover, TP explained the highest proportion of variation in all bacterial community composition (Figure 4B, explained variation = 8.73%). In addition, SEM was used to examine the underlying pathways through which nutrient addition influences both bacterial diversity and community structure (Figure 5A). The SEM showed that increased NH_4_^+^-N after N addition directly induced changes in soil pH and DOC. In addition, the increased AP content after P addition promoted an increase in DOC. The pathways involving NH_4_^+^-N, AP, AK, pH, and DOC together explained 31 and 72% of the total variance in bacterial Shannon diversity and bacterial community structure, respectively. Nutrient addition can alter soil microbial diversity both by the direct impact of changes in soil AP levels and by indirect effects via change of soil DOC. Furthermore, the alteration of nutrient availability (NH_4_^+^-N, AP, and AK) and DOC can affect the soil bacterial community composition. We further investigated the relationships between microbial genera and soil properties in the form of network visualizations (Figure 5B). NH_4_^+^-N (node degree = 20), AP (node degree = 18), pH (node degree = 16), and DOC (node degree = 13) were significantly correlated with more than 10 microbial genera. However, we found that TK and AK were not significantly correlated with any microbial genera.

**Table 2:** Statistical results of Mantel tests and distance-based redundancy analyses (dbRDA) evaluating the effects of soil chemical properties on bacterial community structure.

Explanatory variables	Mantel r-statistic	dbRDA % variation explained
NO_3_^−^-N	0.262*	7.7574*
NH_4_^+^-N	0.241*	9.2158*
TP	0.363***	9.9255*
AP	0.245***	9.2807*
pH	0.368***	13.879*
DOC	0.307**	7.7055*
C/P	0.225**	8.2065*
N/P	0.214**	8.1438*
TN	0.110	3.6349
TK	0.011	3.8085
AK	0.001	3.4426
SOC	−0.002	3.5604
C/N	0.116	2.3649
SWC	0.073	7.1203*

Asterisks indicate the significance level: *p < 0.05, **p < 0.01, and ***p < 0.001.

### 3.2 Effects of nutrient additions on soil functional groups

**Figure 6:**
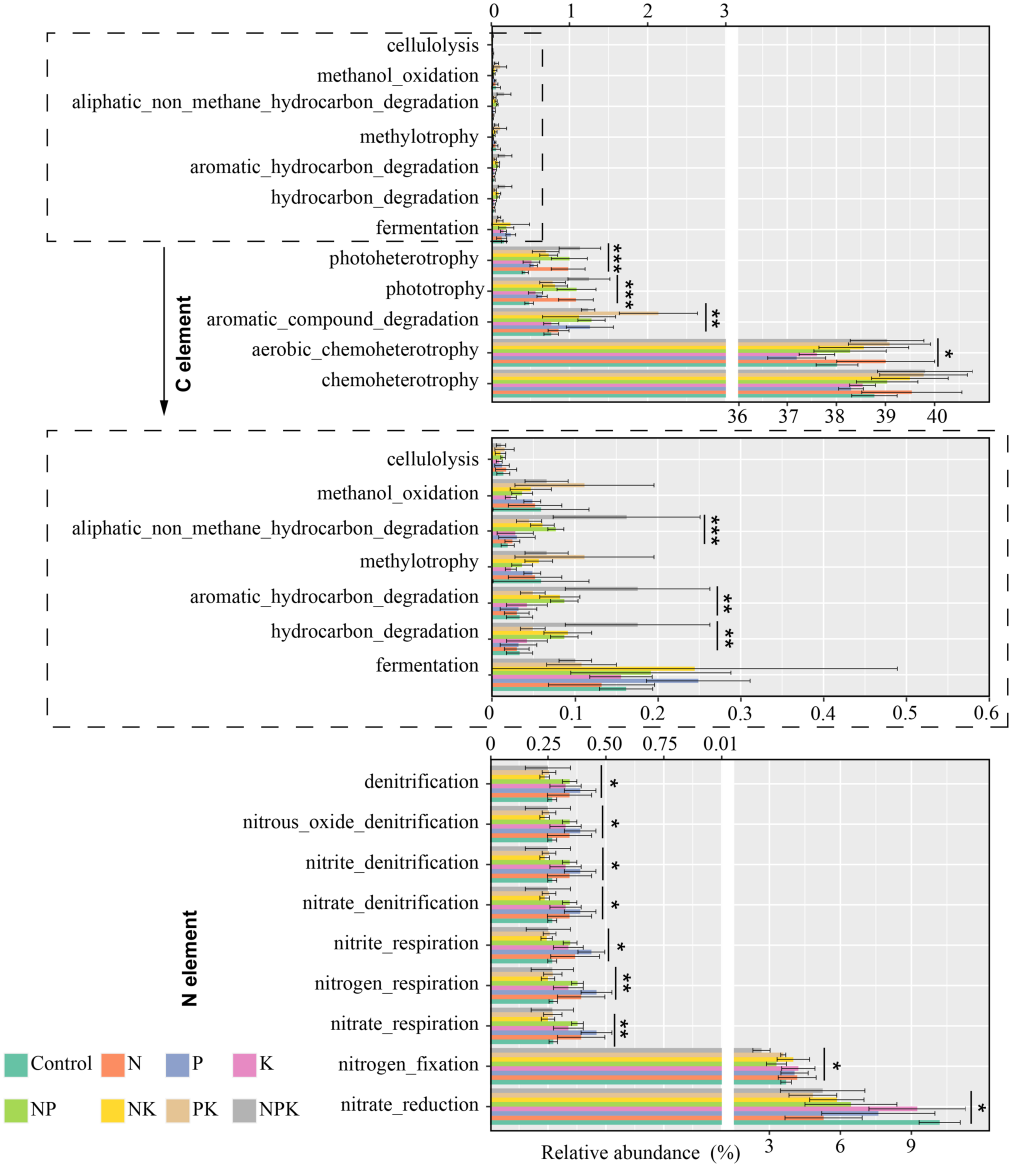
The potential ecological function changes of soil bacterial communities under different nutrient addition treatments. Error bars indicate standard deviations (n = 4). Significance levels: *p < 0.05, **p < 0.01, and ***p < 0.001.

To explore whether and how long-term nutrient addition affects ecosystem community function, FAPROTAX was used to predict microbial functions. The results from FAPROTAX indicated the identification of 42 microbial pathways, with 30 linked to the cycling of C and N. The four most common functional groups were chemoheterotrophy, aerobic chemoheterotrophy, nitrate reduction, and nitrogen fixation, accounting for 39.16, 38.35, 6.84, and 3.72%, respectively. Long-term nutrient addition significantly changed bacterial functions related to the C-cycle, including aerobic chemoheterotrophy, aromatic compound degradation, phototrophy, photoheterotrophy, hydrocarbon degradation, aromatic hydrocarbon degradation, and aliphatic non-methane hydrocarbon degradation (Figure 6). The relative abundance of functional groups involved in chemoheterotrophy, aerobic chemoheterotrophy, phototrophy, and photoheterotrophy increased in the N-only addition treatment. Aromatic compound degradation, phototrophy, photoheterotrophy, and fermentation functional groups increased in the P-only addition treatment, but chemoheterotrophy and aerobic chemoheterotrophy were inhibited. In addition, we noted that multiple additions (NP, NK, PK, and NPK) increased most of the C-cycle functional groups, but the fermentation functional group was inhibited in the PK and NPK addition treatments. Moreover, we observed that a considerable proportion of functional groups involved in N cycling exhibited changes with different nutrient addition treatments. All nutrient addition treatments exhibited varying degrees of reduction in nitrate ester functional groups compared to the control. The nitrate reduction functional group decreased to varying degrees across all nutrient addition treatments. In addition, N-only, P-only, and NP additions improved soil denitrification (Figure 6).

When correlations between soil chemical properties and bacterial functions were explored, it was found that DOC was the most important factor correlated with all C-cycle functional groups, except for cellulolysis. Furthermore, a high correlation existed between most bacterial functional groups of C cycling and AP, NH_4_^+^-N, and SWC. Except for the nitrate reduction and nitrogen fixation groups, no significant relationship was observed between the relative abundance of other bacterial functional groups involved in N cycling and soil chemical properties (Supplementary Figure 1).

## 4 Discussion

### 4.1 Nitrogen and phosphorus addition alters soil bacterial composition

It is well established that soil bacterial communities are extremely sensitive to N addition globally (Dong et al., 2021; Leff et al., 2015). Furthermore, we discovered that P addition can cause significant changes in the bacterial community structure in the soil (Figure 3). Our findings are supported by previous studies in alpine grassland and semi-arid steppe (Chen et al., 2020; Ling et al., 2017). Differences in dominant bacterial taxa can serve as indicators of changes in bacterial communities resulting from nutrient addition (Ling et al., 2017). *Actinobacteria* (~40%) was the most prevalent phylum in the temperate grassland region where our research was conducted, followed by *Proteobacteria* (~25%) and *Acidobacteriota* (~11%). Dai et al. (2018) suggested that N addition is beneficial for the growth of *Actinobacteria*, but it suppresses *Acidobacteriota.* Our findings were partially consistent with this. Since multiple clusters of genera exhibited contrasting responses, *Actinobacteria* did not show a clear pattern in response to N addition (Figure 1B; Supplementary Table 5). Among dominant genera in *Actinobacteria*, N addition resulted in an increase in *Mycobacterium* while decreasing *Rubrobacter*. *Actinobacteria* is recognized as one of the richest bacterial taxa in soil (~22%), thriving in diverse ecological zones (Araujo et al., 2020; Delgado-Baquerizo et al., 2017; Ho et al., 2017). Considering the divergent living strategies in Actinobacterial communities, the different responses of the majority of Actinobacterial taxa to N addition obscured the overall trend. This is supported by the oligotrophic–copiotrophic theory (Fierer et al., 2007; Stone et al., 2023), which demonstrates that N addition promoted the growth of copiotrophic bacteria (*Proteobacteria*, *Firmicutes*, *Bacteroidota*, etc.) but inhibited the growth of oligotrophic bacteria (*Acidobacteria*, *Chloroflexi*, *Verrucomicrobiota*, etc.; Figure 1B). Moreover, changes in bacterial composition at the phylum and dominant genera levels were consistent. P additions did not conform to this pattern, and only *Acidobacteria*, *Firmicutes,* and *Chloroflexi* showed positive responses to P addition. However, the abundances of Actinobacteria and Proteobacteria were negatively impacted by P addition. It has been previously reported that these bacterial phyla contain *phoD* genes (Ikoyi et al., 2018; Jiang et al., 2023; Wang et al., 2023). According to Chen et al. (2019), long-term P addition significantly altered soil microbial communities and reduced the proportion of *phoD*-harboring bacteria. The phenomenon supports the idea that long-term mineral P input inhibits the growth of *phoD*-harboring bacteria. According to the results, many soil bacterial taxa were affected oppositely by N-only and P-only additions at the phylum or genus levels. However, when nitrogen and phosphorus are added together, the divergent changes caused by single additions can be alleviated or counterbalanced. Moreover, compared to N and P additions, it was noted that K additions did not alter soil bacterial communities. Previous research has also indicated that soil microorganisms demand more N and P than K while being less susceptible to potassium deficiency compared to crops (Moro et al., 2014; Paul et al., 2021).

### 4.2 Phosphorus addition rather than nitrogen addition can change soil bacterial alpha diversity

Soil microorganisms serve as important indicators for shaping soil C and N cycling processes. They are typically sensitive to nutrient additions, especially soil bacteria (Dai et al., 2018). This study presents novel findings that contribute to our understanding of how nutrient additions impact bacterial communities in temperate steppe environments. The α-diversity of bacteria displayed a positive response under N and P addition treatments. *Mounting* evidence suggests that N enrichment can result in a reduction in bacterial α-diversity (Dai et al., 2018; Elser et al., 2007; Fierer et al., 2012). It was discovered in the study that N addition had no significant effect on multiple α-diversity indices of soil bacteria (Table 1). This could be due to the fact that bacterial α-diversity may not exhibit a significant decreasing trend before reaching the critical threshold of nitrogen enrichment (Scheffer et al., 2012; Zhou et al., 2020).

According to research by Zeng et al. (2016) and Liu et al. (2020) on typical temperate steppes, the response thresholds of soil bacterial diversity to nitrogen enrichment were found to be 12 g-N m^−2^ yr.^−1^ and 16 g-N m^−2^ yr.^−1^, respectively. Furthermore, Dong et al. (2021) found the threshold for N enrichment at 30 g-N m^−2^ yr.^−1^ in the meadow steppes. Despite observing no significant negative impact on bacterial α-diversity from N-only addition, the Chao1, Shannon, and PD indices showed a great promotion when N and P were added together. However, the bacterial α-diversity did not change when N was applied with K. These varying results indicate that the effects of different nutrient addition treatments on soil bacteria were inconsistent, potentially influenced by specific environmental conditions and soil nutrient status (Fang et al., 2023).

Increasing evidence has demonstrated that the alterations in soil pH and nutrient content resulting from nutrient addition are crucial parameters in changing soil bacterial α-diversity and community composition (Fierer and Jackson, 2006; Hou et al., 2021). A previous meta-analysis suggested that N addition primarily shapes bacterial α-diversity by altering soil pH (Zhou et al., 2020). Soil pH directly limits or constrains the physiological activities of microorganisms, and exceeding a certain range (i.e., ecological niche) leads to a decrease in the net growth of individual taxa that are incapable of surviving under such conditions (Zhou et al., 2020). Bacteria generally exhibit optimal growth at neutral pH (Fierer and Jackson, 2006; Zhou et al., 2020). The pH values of soil samples in the study area were found to be neutral to slightly alkaline. Despite the fact that soil acidification can occur with single-N addition, the additions of NP, NK, and NPK did not significantly affect soil pH (Supplementary Table 1). Therefore, slight fluctuations in soil pH values may not result in significant changes in soil bacterial communities, contrary to our initial hypothesis. Soil bacterial diversity is more sensitive to P addition than N addition. Moreover, bacterial diversity indices including Shannon and PD did not correlate with soil pH but showed a significant correlation with available P, C/P, and N/P. This may be because phosphorus plays a crucial role in the normal growth and metabolic processes of living organisms, participating in various biological processes, including the synthesis of nucleotides and the regulation of enzyme activity (Elser et al., 2003; Peñuelas and Sardans, 2009; Xia et al., 2023). As rapidly growing organisms, bacteria have a higher demand for phosphorus. Therefore, changes in bacterial diversity as a result of P addition could be attributed to the alleviation of microbial phosphorus limitation caused by increased phosphorus input, promoting the growth of the microbial community (Cui et al., 2022). This further indicates that soil P availability is potentially the major factor influencing bacterial diversity under nutrient addition.

### 4.3 Nitrogen and phosphorus addition alters the structure and function of soil bacterial communities

The growth and metabolic activities of microorganisms are influenced by various environmental factors (Wang et al., 2017; Xiao et al., 2022). Our study suggested that the most significant influences on the structure of bacterial communities were found to be available nitrogen, phosphorus content, pH, and DOC, as validated by both RDA and Mantel analyses. This finding is consistent with our second hypothesis. A previous study indicated that N addition led to an average decrease of 0.16 units in soil pH across 25 grassland sites (Leff et al., 2015). However, pH might not be the only factor driving community changes in our study. Evidence from SEM findings suggested that pH had a limited influence on bacterial community transformations under N addition (Figure 5A). In addition, numerous significant correlations were observed between soil-available nutrients (e.g., NH_4_^+^-N, AP, and DOC) and bacterial genera based on the correlation network analysis in this study. These results suggested that bacterial community composition was not only strongly altered by soil pH but also regulated by nutrient availability under long-term N and P addition. Our findings are consistent with those of Zhou et al. (2017), suggesting that N addition mainly affects microbial communities by enhancing resources instead of acidifying the soil. Meanwhile, increasing P availability through exogenous P addition has resulted in changes to the soil bacterial communities. Moreover, both N and P additions led to an increase in DOC (Supplementary Table 1), suggesting that the accelerated mineralization of soil carbon through N and P additions can provide additional energy and nutrients for soil microorganisms. The alteration of nutrient availability and DOC can affect soil bacterial community structure (Kaspari et al., 2017; Philippot et al., 2013; Wang et al., 2018b).

The alterations in bacterial communities observed during long-term nutrient addition, by extension, lead to shifts in microbial metabolic functions (Wang et al., 2017; Xiao et al., 2022). Chemoheterotrophy is the primary pathway of carbon flow in microbial communities. N addition treatments (only-N, NP, NK, and NPK) increased the chemoautotrophy functional group, while the only-P addition treatment had a negative effect on it (Figure 6). Chen et al. (2021) also found that the process of decomposing unstable and recalcitrant organic matter was significantly enhanced with increased N addition. In addition, unstable organic carbon showed a more sensitive response to N addition. Moreover, the negative response of *Actinobacteria* and *Proteobacteria* to P addition may be the cause of the weakening of the chemoheterotrophy functional group as soil organic matter decomposition is highly dependent on *Proteobacteria* and *Actinobacteria* (Dai et al., 2018). Furthermore, our research found that the phototrophy, aromatic compound degradation, and fermentation functional groups increased in the P-only addition treatment (Figure 6). The increase in aromatic compound degradation and fermentation functional groups indicates that P addition can enhance the ability of soil to degrade complex organic compounds. However, the increase in phototrophy functional groups can contribute to the accumulation of soil organic carbon (Sun et al., 2023).

Microorganisms are crucial contributors to nitrogen-fixing and transforming processes in soil (Lin et al., 2023). N-only, P-only, and NP additions improved soil denitrification (Figure 6). The increased abundance of denitrifiers under nutrient-rich conditions is likely attributed to the fact that many of them are copiotrophs, which prefer such conditions for growth (Che et al., 2018; Liu et al., 2012; Morrissey and Franklin, 2015). Furthermore, nutrient addition could lead to increased anaerobic environments through enhanced microbial respiration (Liu et al., 2012; Lu et al., 2011), thereby indirectly stimulating the expression of denitrification genes. Moreover, denitrifiers are influenced by many environmental factors (Wallenstein et al., 2006), and previous studies have revealed inconsistent responses of denitrification genes to N and P additions (Tang et al., 2016; Wang et al., 2019). This discrepancy indicated that denitrification-relevant gene expression might be linked to ecosystem types and environmental factors (Tang et al., 2016; Wallenstein et al., 2006). However, it should be noted that current functional gene annotation tools based on 16S rRNA gene amplicons usually have limited predictive power due to the unculturable nature of most bacteria in soils (Louca et al., 2019). Therefore, more accurate methods are essential to enhance the precision of functional profiles in future studies. In brief, our findings emphasized the importance of soil phosphorus in determining bacterial communities in temperate steppe. Numerous studies have primarily focused on how nitrogen deposition affects regions globally (Hu et al., 2021; Nie et al., 2019; Sun et al., 2023; Zhou et al., 2017), while research on phosphorus addition has mainly focused on phosphorus-limited tropical forests (O'Neill et al., 2022; Wang et al., 2018b). Therefore, it is of critical importance to conduct further systematic research on P addition on soil microbial involvement in C and N cycling in grassland ecosystems. Furthermore, long-term studies incorporating different rates and frequencies of phosphorus addition would enhance our understanding of its impact on soil microbial communities.

## 5 Conclusion

Soil bacterial diversity exhibited differential responses to the addition of N and P in a steppe meadow. Soil bacterial α-diversity was more sensitive to P addition than to N addition, primarily due to alterations in soil P availability resulting from P addition. Moreover, 11 years of nutrient addition shifted the structure and function of soil bacterial communities. Soil bacterial communities are altered more by soil resource enhancement (e.g., nutrient availability and DOC) than pH following long-term N and P addition. In addition, nutrient addition may indirectly impact the functions of C and N cycling in microorganisms by changing nutrient availability in the soil. N addition treatments (only-N, NP, NK, and NPK) increased the chemoautotrophy functional group, while P-only addition treatment increased the phototrophy, aromatic compound degradation, and fermentation functional groups. Furthermore, N-only, P-only, and NP additions enhanced soil denitrification. Overall, these findings suggest that long-term phosphorus addition significantly affects soil microbial communities that regulate key C and N cycling processes, providing fresh perspectives on the response of meadow ecosystems to nutrient additions.

## Data Availability

The original contributions presented in the study are included in the article/Supplementary material; further inquiries can be directed to the corresponding authors. The names of the repository/repositories and accession number(s) can be found at: https://www.ncbi.nlm.nih.gov/, PRJNA928583.

## Footnotes

^1^https://report.majorbio.com/meta/FAPROTAX/
