# Supplementary material for: Soil bacterial community composition is altered more by soil nutrient availability than pH following long-term nutrient addition in a temperate steppe
Source: Front Microbiol. 2024 Sep 13;15:1455891. doi: 10.3389/fmicb.2024.1455891 (PMC11427344; doi:10.3389/fmicb.2024.1455891)
Supplement: Supplementary file 1 [file Data_Sheet_1.pdf]

Supplementary Material

1 Supplementary Figures and Tables

1.1 Supplementary Tables

Supplementary Table 1 Soil physical and chemical properties in different nutrient additions

Analysis index	Control	N	P	K	NP	NK	PK	NPK
SOC (g/kg)	38.3±2.18a	39.05±3.77a	38.08±2.66a	36.9±1.72a	37.83±1.55a	37.58±1.37a	34.73±1.69a	36.95±1.38a
pH	6.48±0.1a	5.76±0.33b	6.38±0.08ab	6.51±0.26a	5.97±0.26ab	6.02±0.48ab	6.17±0.14ab	5.97±0.43ab
TK (g/kg)	6.06±0.01bc	6.06±0.03bc	6.08±0.01bc	6.92±0.75a	5.83±0.27c	6.42±0.27abc	6.56±0.05ab	5.95±0.25bc
AK (g/kg)	0.21±0.03c	0.2±0.03c	0.22±0.02c	0.99±0.12a	0.15±0.03c	0.74±0.1b	0.77±0.06b	0.58±0.14b
TN (g/kg)	3.49±0.18a	3.58±0.24a	3.35±0.14a	3.46±0.11a	3.4±0.14a	3.42±0.16a	3.14±0.36a	3.36±0.18a
TP (g/kg)	0.4±0.02b	0.37±0.02b	0.91±0.07a	0.39±0.01b	1.02±0.11a	0.37±0.01b	1.13±0.19a	1.00±0.14a
AP (mg/kg)	6.23±1.3c	6.73±0.25c	147.28±22.51b	6.97±0.87c	193.5±37.96a	6.73±0.71c	190.83±20.28ab	167.33±26.55ab
NH_4^+-N (mg/kg)	5.25±0.6b	14.71±5.19a	6.96±0.7ab	5.97±2.15b	10.92±7.51ab	9.67±4.03ab	7.7±0.9ab	6.94±1.45ab
NO_3^--N (mg/kg)	20.86±2.14abc	40±17.58a	13.39±4.13bc	17.61±1.39bc	26.64±8.2abc	28.94±13.71ab	7.08±0.88c	27.92±9.71abc
DOC (mg/kg)	484.68±54.18a	519.36±37.75a	507.21±49.73a	445.89±19.98a	528.09±148.66a	518.59±44.66a	587.32±25.29a	600.28±57.13a
SWC (%)	20.84±1.31a	19.3±2.8a	21.06±0.7a	21.13±1.3a	18.91±0.99a	20.87±0.92a	19.58±0.67a	19.29±0.93a
C/N	10.98±0.43a	10.9±0.33a	11.36±0.44a	10.67±0.74a	11.14±0.42a	11±0.55a	11.13±0.85a	11.01±0.58a
C/P	95.64±7.53ab	105.85±5.25a	41.76±3.01c	94.1±3.16b	37.56±4.77c	101.34±2.73ab	31.26±3.96c	37.57±5.9c
N/P	8.72±0.71a	9.7±0.2a	3.68±0.3b	8.83±0.37a	3.36±0.32b	9.23±0.56a	2.82±0.39b	3.41±0.51b

Different labeled letters indicate significant differences between different treatments according to one-way ANOVA with Duncan's multiple range tests ($P < 0.05$).

Supplementary Table 2 Soil physical and chemical properties in different nutrient additions

	N		P		K		N: P		N: K		P: K		N: P: K	
	F	*P*	*F*	*P*	*F*	*P*	*F*	*P*	*F*	*P*	*F*	*P*	*F*	*P*
observed_species	3.039	0.094	**6.425**	**0.018**	0.002	0.961	0.706	0.409	2.602	0.120	**5.076**	**0.034**	0.068	0.797
Chao1	2.688	0.114	2.462	0.130	0.015	0.904	0.598	0.447	**4.413**	**0.046**	3.594	0.070	0.956	0.338
Shannon	0.000	0.994	**8.041**	**0.009**	0.045	0.835	0.273	0.606	0.625	0.437	2.910	0.101	0.023	0.880
PD_whole_tree	2.518	0.126	**9.582**	**0.005**	0.003	0.954	1.232	0.278	3.915	0.059	**4.956**	**0.036**	0.144	0.707

The bold numbers are significant values.

Supplementary Table 3 The significant differences test in soil bacterial α-diversity, phylum-level abundance, and genus-level abundance between single and Multiple addition

α-diversity,	*F*	*P*	phylum-level	*F*	*P*	genus-level	*F*	*P*
observed_species	0.060	0.808	p__Myxococcota	2.835	0.103	g_Bradyrhizobium	**5.380**	**0.027**
Chao1	0.267	0.609	p__Patescibacteria	**4.876**	**0.035**	g_Candidatus_Udaeobacter	0.167	0.686
Shannon	1.713	0.200	p__Bacteroidota	**14.997**	**0.001**	g_norank_f_Xanthobacteraceae	**13.225**	**0.001**
PD_whole_tree	0.230	0.635	p__Gemmatimonadota	3.035	0.092	g_RB41	**14.246**	**0.001**
			p__Firmicutes	0.284	0.598	g_norank_ o_Vicinamibacterales	1.658	0.208
			p__Verrucomicrobiota	0.199	0.659	g_Mycobacterium	0.334	0.568
			p__Chloroflexi	0.284	0.598	g_norank_ o_Gaiellales	0.141	0.710
			p__Acidobacteriota	4.134	0.051	g_Rubrobacter	**13.442**	**0.001**
			p__Proteobacteria	0.361	0.553	g_Sphingomonas	**4.329**	**0.046**
			p__Actinobacteriota	0.008	0.930	g_norank_f_67-14	4.042	0.053

The bold numbers are significant values.

Supplementary Table 4 The correlation analysis between soil α-diversity and environmental factors

	TP	AP	NO_3^--N	C/P	N/P
observed_species	0.330	0.315	-0.415*	-0.362*	-0.340
Chao1	0.169	0.100	-0.355*	-0.194	-0.178
Shannon	0.422*	0.463**	-0.206	-0.477**	-0.450**
PD_whole_tree	0.342	0.353*	-0.404*	-0.389*	-0.378*

Asterisks indicate the significance level: "*" P < 0.05, "**" P < 0.01.

Supplementary Table 5 Three-way ANOVA results testing effects of different nutrient additions on phylum-level abundance

	N		P		K		N: P		N: K		P: K		N: P: K	
	F	*P*	*F*	*P*	*F*	*P*	*F*	*P*	*F*	*P*	*F*	*P*	*F*	*P*
p__Myxococcota	0.030	0.865	**7.435**	**0.012**	2.148	0.156	1.454	0.240	0.444	0.512	0.145	0.707	0.676	0.419
p__Patescibacteria	**6.788**	**0.016**	0.056	0.814	1.589	0.220	3.218	0.085	0.005	0.947	0.101	0.753	0.797	0.381
p__Bacteroidota	**8.222**	**0.008**	2.955	0.098	**5.629**	**.026**	3.914	0.059	1.240	0.277	2.696	0.114	1.481	0.235
p__Gemmatimonadota	**6.837**	**0.015**	0.195	0.663	0.001	0.980	0.009	0.927	**5.887**	**0.023**	0.830	0.371	0.518	0.479
p__Firmicutes	0.006	0.941	0.025	0.875	0.758	0.392	**4.681**	**0.041**	2.517	0.126	1.685	0.207	0.000	0.985
p__Verrucomicrobiota	0.041	0.841	0.034	0.856	0.074	0.788	0.157	0.695	3.721	0.066	0.633	0.434	0.996	0.328
p__Chloroflexi	**18.453**	**0.000**	**12.963**	**0.001**	0.783	0.385	2.427	0.132	0.406	0.530	3.339	0.080	0.003	0.960
p__Acidobacteriota	**12.414**	**0.002**	1.205	0.283	**6.323**	**0.019**	1.492	0.234	3.401	0.078	**8.601**	**0.007**	0.116	0.737
p__Proteobacteria	**4.748**	**0.039**	1.147	0.295	0.238	0.630	0.562	0.461	1.354	0.256	2.082	0.162	0.102	0.752
p__Actinobacteriota	0.147	0.705	1.939	0.177	0.243	0.627	1.849	0.187	0.215	0.647	1.554	0.225	0.482	0.494

The bold numbers are significant values.

Supplementary Table 6 Three-way ANOVA results testing effects of different nutrient additions on genus-level abundance

	N		P		K		N: P		N: K		P: K		N: P: K	
	F	*P*	*F*	*P*	*F*	*P*	*F*	*P*	*F*	*P*	*F*	*P*	*F*	*P*
g__Bradyrhizobium	2.367	0.137	**8.569**	**0.007**	0.124	0.728	**5.096**	**0.033**	1.549	0.225	2.161	0.155	0.126	0.725
g_Candidatus_Udaeobacter	0.032	0.859	0.025	0.876	0.054	0.819	0.182	0.674	3.935	0.059	0.643	0.430	0.838	0.369
g_norank_f_Xanthobacteraceae	0.027	0.872	**17.019**	**<0.001**	**5.524**	**0.027**	0.469	0.500	0.000	0.986	0.040	0.843	1.078	0.310
g_RB41	**27.622**	**<0.001**	0.049	0.828	**12.343**	**0.002**	0.241	0.628	**5.576**	**0.027**	**8.800**	**0.007**	1.345	0.258
g_norank_o_Vicinamibacterales	**6.687**	**0.016**	1.863	0.185	**6.380**	**0.019**	2.493	0.127	2.699	0.113	**8.298**	**0.008**	0.183	0.673
g__Mycobacterium	**7.211**	**0.013**	2.121	0.158	0.004	0.952	**5.983**	**0.022**	0.177	0.677	0.152	0.700	0.024	0.879
g_norank_o_Gaiellales	0.794	0.382	**8.743**	**0.007**	0.382	0.542	**7.030**	**0.014**	0.004	0.950	1.476	0.236	3.105	0.091
g_Rubrobacter	**19.207**	**<0.001**	**8.711**	**0.007**	3.671	0.067	**12.372**	**0.002**	1.263	0.272	1.506	0.232	0.012	0.915
g_Sphingomonas	2.808	0.107	1.652	0.211	**5.003**	**0.035**	0.204	0.655	0.023	0.881	**6.708**	**0.016**	0.435	0.516
g_norank_f_67-14	0.262	0.614	**11.275**	**0.003**	0.780	0.386	1.156	0.293	0.512	0.481	0.187	0.669	0.232	0.634

The bold numbers are significant values.

1.2 Supplementary Figures

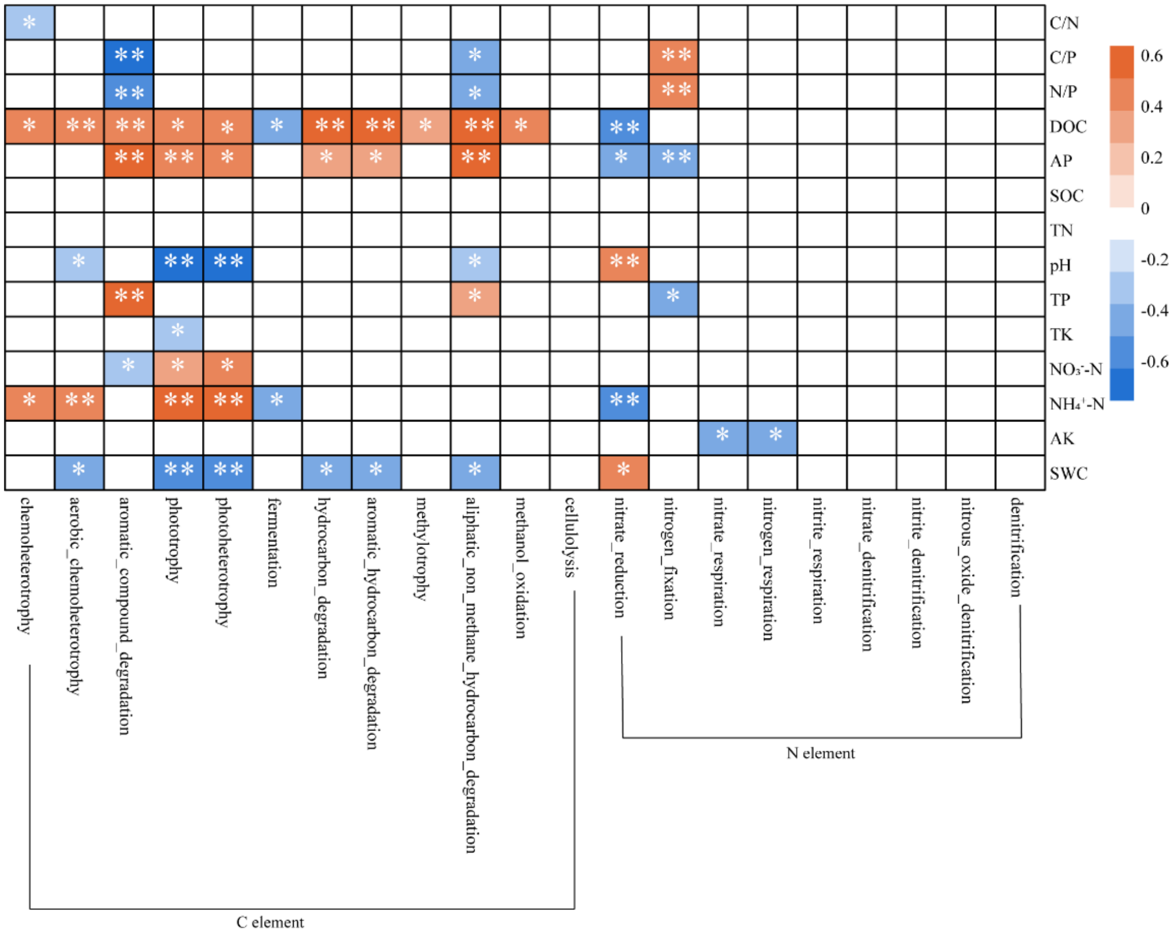

Supplementary Figure 1. The correlation analysis between functional groups and environmental factors. Asterisks indicate the significance level: "*" $P < 0.05$, "**" $P < 0.01$, "***" $P < 0.001$.
